# The NitroSpeed Taniborbactam NP test as a rapid test for detection of β-lactamase-mediated susceptibility to taniborbactam

**DOI:** 10.1093/femsle/fnaf044

**Published:** 2025-05-06

**Authors:** Otávio Hallal Ferreira Raro, Soraya Herrera-Espejo, Maxime Bouvier, Auriane Kerbol, Laurent Poirel, Patrice Nordmann

**Affiliations:** Medical and Molecular Microbiology, Faculty of Science and Medicine, University of Fribourg, CH-1700 Fribourg, Switzerland; Medical and Molecular Microbiology, Faculty of Science and Medicine, University of Fribourg, CH-1700 Fribourg, Switzerland; Clinical Unit of Infectious Diseases, Microbiology and Parasitology, Institute of Biomedicine of Seville (IBiS), Virgen del Rocio University Hospital/CSIC/University of Seville, 41013 Seville, Spain; Medical and Molecular Microbiology, Faculty of Science and Medicine, University of Fribourg, CH-1700 Fribourg, Switzerland; Swiss National Reference Center for Emerging Antibiotic Resistance (NARA), University of Fribourg, CH-1700 Fribourg, Switzerland; Medical and Molecular Microbiology, Faculty of Science and Medicine, University of Fribourg, CH-1700 Fribourg, Switzerland; Swiss National Reference Center for Emerging Antibiotic Resistance (NARA), University of Fribourg, CH-1700 Fribourg, Switzerland; Medical and Molecular Microbiology, Faculty of Science and Medicine, University of Fribourg, CH-1700 Fribourg, Switzerland; Swiss National Reference Center for Emerging Antibiotic Resistance (NARA), University of Fribourg, CH-1700 Fribourg, Switzerland; Medical and Molecular Microbiology, Faculty of Science and Medicine, University of Fribourg, CH-1700 Fribourg, Switzerland; Swiss National Reference Center for Emerging Antibiotic Resistance (NARA), University of Fribourg, CH-1700 Fribourg, Switzerland

**Keywords:** Enterobacterales, *P. aeruginosa*, metallo-β-lactamases, β-lactamase inhibitors, taniborbactam, NitroSpeed Taniborbactam NP test

## Abstract

Taniborbactam (TAN) is an investigational β-lactamase inhibitor in clinical development combined with cefepime for the treatment of bacterial infections caused by broad-spectrum β-lactamase-expressing bacteria. Its spectrum of inhibition encompasses all classes of β-lactamases, including clinically important metallo-β-lactamases (MBLs) NDM-1 and VIM-2. However, TAN lacks a significant inhibition of imipenemase-type β-lactamases. Rare TAN-resistant New Delhi metallo-β-lactamase (NDM) or Verona integron-encoded metallo-β-lactamase (VIM) variants (namely NDM-9, NDM-30, and VIM-83) have been identified. The NitroSpeed Taniborbactam NP test was developed to rapidly assess the β-lactamase inhibitory activity of TAN against various β-lactamases, particularly serving as an efficient tool for identifying compounds with potential activity against different types of MBLs. The test is based on the hydrolysis of (i) nitrocefin (to determine the presence or absence of β-lactamase), (ii) ertapenem (to confirm the presence or the absence of carbapenemase), and (iii) TAN (to assess whether the carbapenemase is inhibited by TAN). The test was validated using a collection of 134 genetically characterized clinical isolates (103 Enterobacterales and 31 *Pseudomonas aeruginosa*). The NitroSpeed Taniborbactam NP test is simple, easy to perform, and provides results within ≤15 min. When evaluated against a broad set of β-lactamases, the test demonstrated 100% sensitivity, specificity, and accuracy.

## Teaser

The NitroSpeed Taniborbactam NP test is a tool for evaluating metallo-β-lactamase inhibition by taniborbactam.

## Introduction

Metallo-β-lactamases (MBLs) are zinc-dependent enzymes classified as class B, following the Ambler β-lactamase classification (Ambler 1980). These enzymes hydrolyse β-lactams from all classes except monobactams. MBLs are divided into three subclasses—B1, B2, and B3—based on differences in amino acid sequences at several structural sites, including active sites, loop structures, substrate affinity, zinc content, and ligand interactions (Bush 2013, López et al. 2022).

The most clinically significant MBLs belong to class B1, including New Delhi metallo-β-lactamase (NDM) and Verona integron-encoded metallo-β-lactamase (VIM). The corresponding genes are predominantly plasmid-borne [e.g NDM, VIM, imipenemase (IMP)], and are disseminated across multiple Gram-negative bacterial species (López et al. 2022). Subclass B2 is represented by the chromosomally encoded CphA enzyme, which occurs naturally in *Aeromonas* spp. (Segatore et al. 1993). Subclass B3 includes the intrinsic, chromosomally encoded MBLs L1 and GOB from *Stenotrophomonas maltophilia* and *Elizabethkingia meningoseptica*, respectively (Akova et al. 1991, González and Vila 2012, Boyd et al. 2020), as well as the plasmid-encoded *Serratia* MBL and Adelaide IMP enzymes (Wachino et al. 2011, Zhou et al. 2019).

MBL-encoding genes are highly transferable, and variants with resistance mutate at an alarming rate, especially in the absence of clinically available MBL inhibitors (Tooke et al. 2019, Bush and Bradford 2020). Currently, clinically available β-lactamase inhibitors target class A, C, and D β-lactamases, including KPC and OXA-48-like enzymes. Avibactam is active against class A, C, and D β-lactamases, while vaborbactam and relebactam are active against class A and C β-lactamases (Abboud et al. 2016, Lomovskaya et al. 2017, Campanella and Gallagher 2020). The aztreonam–avibactam (AZA) combination is effective against most MBL-producing bacteria since MBLs do not hydrolyse aztreonam and avibactam inhibits the activity of class A and D β-lactamases, preserving the aztreonam’s efficacy. However, AZA is inactive against *Acinetobacter baumannii in vitro* due to intrinsic resistance mechanisms such as efflux pumps and AmpC overproduction. Resistance to AZA has been reported in some Enterobacterales, mainly due to alterations in penicillin-binding protein 3, the target of aztreonam (Yahav et al. 2020).

MBL inhibitors currently in development include the third-generation boronate compounds such as taniborbactam (TAN) and xeruborbactam. TAN is in phase 3 clinical trials in combination with cefepime (Yahav et al. 2020). While it effectively inhibits most of the clinically significant NDM- and VIM-type MBLs, TAN has limited activity against IMP-like enzymes (Biedenbach et al. 2015, Lomovskaya et al. 2023). Additionally, NDM-9, expressed by Enterobacterales and *A. baumannii*, is not inhibited by TAN, and resistance to FEP (cefepime)–TAN has already been described in clinical strains from Italy, Switzerland, and the USA, as well as in environmental sources (water) in South Korea (Le Terrier et al. 2023). Similar to NDM-9, the NDM-30 and VIM-83 variants differ from NDM-1 and VIM-1, respectively, by a single amino acid substitution, which confers resistance to TAN inhibition (Tamma and Munita 2024).

To provide guidance on the use of TAN-containing combinations before their widespread implementation, we propose a technique that can detect enzymatic susceptibility or resistance to TAN. This tool may also be useful for screening new compounds with inhibitory activity against MBLs that are not susceptible to TAN inhibition.

## Materials and methods

### Bacterial strains

A collection of 134 clinical isolates from the Medical and Molecular Microbiology Laboratory at the University of Fribourg, Switzerland, was included in this study. This collection comprises 103 Enterobacterales, including *Escherichia coli, Citrobacter freundii, Klebsiella pneumoniae, K. oxytoca, K. variicola, Enterobacter cloacae, E. hormaechei*, and *Serratia marcescens*, as well as 31 *Pseudomonas aeruginosa* genetically characterized isolates. Since our focus was to analyse the efficacy of the MBL inhibitor TAN, we included 115 MBL-producing isolates, 4 of which co-producing a class D carbapenemase. The MBL-producing isolates consisted of 49 NDM-like (36.6%), 31 VIM-like (23.1%), 29 IMP-like (21.6%), a single SPM-1, a single SIM-1 (0.7% each), and 4 NDM + OXA-48-like (3.0%) producers. Among the non-MBL-producing isolates, we tested two KPC-2 (1.5%), a single OXA-48 (0.7%), a single GES-5 (0.7%), four VEB-like producers (3%), three CTX-M-like (2.2%), two TEM-1 (1.5%), a single *E. coli* with AmpC overproduction (0.7%), two *P. aeruginosa* with AmpC overproduction (1.5%), and one isolate each for the reference strains *E. coli* ATCC 25922, *P. aeruginosa* ATCC 27853, and *P. aeruginosa* PAO1 (0.7% each) (Table 1, Supplementary Table 1).

**Table 1.** NitroSpeed Taniborbactam NP test for the detection of β-lactamase enzymatic activity inhibited or not by TAN in Enterobacterales and *P. aeruginosa*.

			NitroSpeed Taniborbactam NP test		
Species	Main acquired β-lactamase (number of isolates)	Ambler class	Water	ETP	ETP + TAN
Enterobacterales	NA (1)	NA	Y	Y	Y
	AmpC overproduction (1)	NA	R	Y	Y
	CTX-M-1 (1)	A	R	Y	Y
	CTX-M-15 (2)	A	R	Y	Y
	TEM-1 (2)	A	R	Y	Y
	VEB-1 (2)	A	R	Y	Y
	KPC-2 (1)	A	R	R	Y
	NDM-1 (20)	B	R	R	Y
	NDM-1 + OXA-48 (2)	B + D	R	R	Y
	NDM-1 + OXA-181 (1)	B + D	R	R	Y
	NDM-4 (2)	B	R	R	Y
	NDM-5 (7)	B	R	R	Y
	NDM-5 + OXA-181 (1)	B + D	R	R	Y
	NDM-7 (4)	B	R	R	Y
	**NDM-9 (7)**	B	**R**	**R**	**R**
	NDM-19 (1)	B	R	R	Y
	**NDM-30** (1)	B	**R**	**R**	**R**
	VIM-1 (14)	B	R	R	Y
	VIM-2 (8)	B	R	R	Y
	VIM-4 (1)	B	R	R	Y
	**VIM-83** (1)	B	**R**	**R**	**R**
	**IMP-1** (3)	B	**R**	**R**	**R**
	**IMP-4 (4)**	B	**R**	**R**	**R**
	**IMP-6 (3)**	B	**R**	**R**	**R**
	**IMP-8 (3)**	B	**R**	**R**	**R**
	**IMP-14 (3)**	B	**R**	**R**	**R**
	**IMP-26 (2)**	B	**R**	**R**	**R**
	**IMP-34 (1)**	B	**R**	**R**	**R**
	**IMP-59 (2)**	B	**R**	**R**	**R**
	**SIM-1 (1)**	B	**R**	**R**	**R**
	OXA-48 (1)	D	R	R	Y
*P. aeruginosa*	NA (2)	NA	Y	Y	Y
	PDC overproduction (2)	NA	R	Y	Y
	GES-5 (1)	A	R	R	Y
	VEB-1 (1)	A	R	Y	Y
	VEB-9 (1)	A	R	Y	Y
	KPC-2 (1)	A	R	R	Y
	NDM-1 (7)	B	R	R	Y
	VIM-1 (1)	B	R	R	Y
	VIM-2 (1)	B	R	R	Y
	VIM-4 (2)	B	R	R	Y
	VIM-5 (2)	B	R	R	Y
	VIM-36 (1)	B	R	R	Y
	**IMP-1 (5)**	B	**R**	**R**	**R**
	**IMP-7 (2)**	B	**R**	**R**	**R**
	**IMP-13 (1)**	B	**R**	**R**	**R**
	SPM-1 (1)	B	R	R	Y

NA, not applicable; Y, yellow colour: enzyme is inhibited by TAN; R, red colour: enzyme is not inhibited by TAN. Bold script indicates enzymes not inhibited by taniborbactam.

### The NitroSpeed Taniborbactam NP test

**Figure 1.**
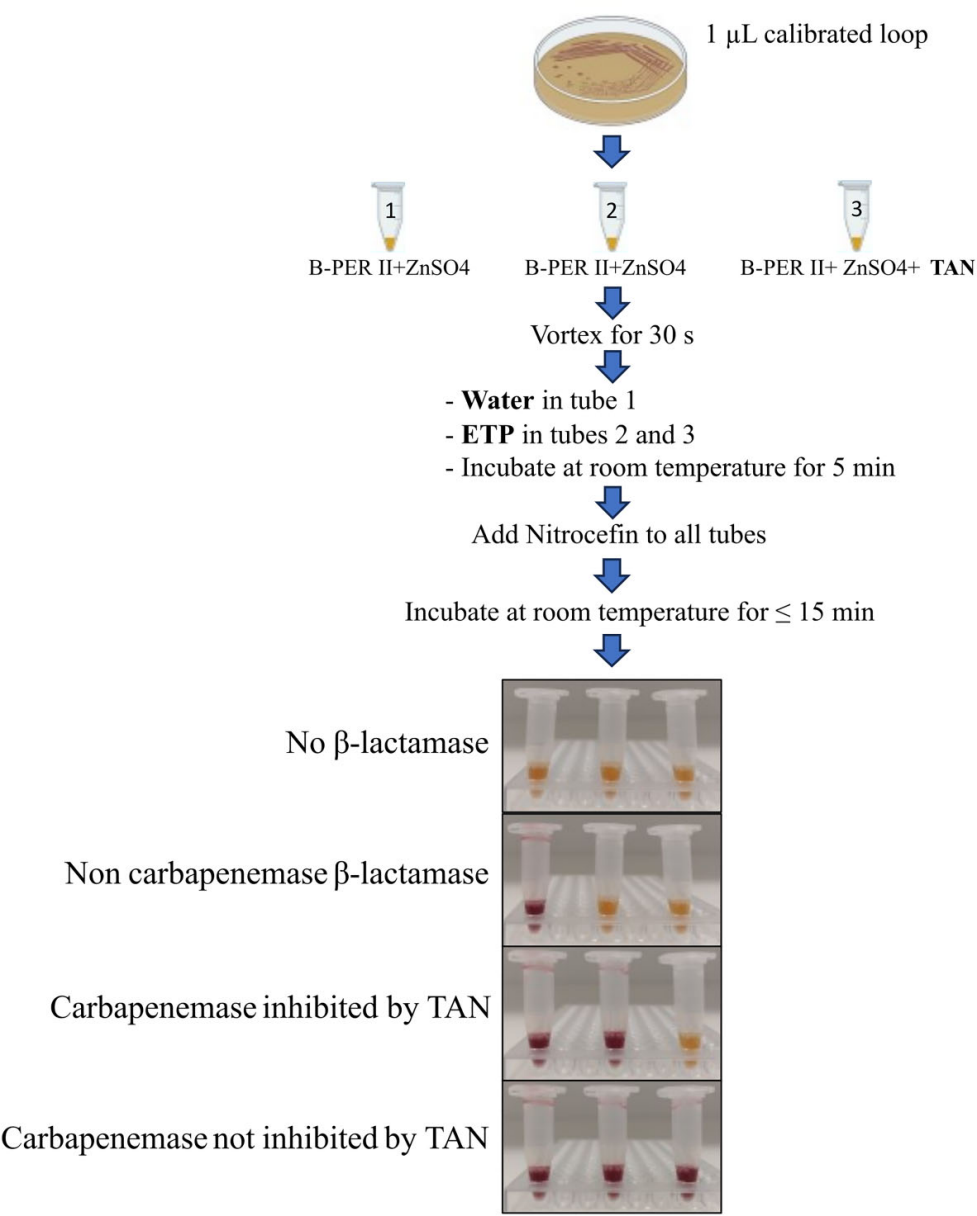
Detailed protocol of the NitroSpeed Taniborbactam NP test. TAN, taniborbactam; ETP, ertapenem; MβL, metallo-β-lactamase. Yellow colour: enzyme is inhibited by TAN; red colour: enzyme is not inhibited by TAN.

The NitroSpeed Taniborbactam test was developed based on the NitroSpeed-Carba NP test (Nordmann et al. 2020). Briefly, isolates were pre-cultivated overnight at 37°C on UriSelect 4 agar plates (Bio-Rad, Marnes-la-Coquette, France). A full 1-µl calibrated loop of each isolate was used as the standard inoculum. Enzymes expressed by the isolates were released by resuspending the colonies in microtubes containing (i) 100 µl of Tris–HCl 20 mM lysis buffer (B-PER II bacterial protein extraction reagent; Thermo Fisher Scientific, IL, USA) with 0.1 mM ZnSO_4_ (Carl Roth) in microtubes 1 and 2; and (ii) 100 µl of Tris–HCl 20 mM lysis buffer with 0.1 mM ZnSO_4_ and 8 mg/l of TAN (MedChemExpress, NJ, USA) in tube 3. After that, microtubes were vortexed for 30 s. Once homogeneous suspensions were obtained, 50 µl of sterile distilled water was added to tube 1, and 50 µl of ertapenem sodium (80 mg/l, Sigma–Aldrich, MO, USA) was added to microtubes 2 and 3 to reach a final concentration of 20 mg/l. After 5 min at room temperature incubation, 50 µl of 1 g/l nitrocefin (Toronto Research Chemicals, Canada) was added to all the microtubes. A detailed protocol of the NitroSpeed Taniborbactam NP test is shown in Fig. 1.

The interpretation of the test was considered optimal when (i) negative controls *E. coli* ATCC 25922 and *P. aeruginosa* ATCC 27853 were negative in all the three tubes, confirming that there was either low-level (basal) or no production of β-lactamase and nitrocefin was not hydrolysed; hence, suspensions remained yellow, (ii) isolates producing a β-lactamase but not carbapenemase were positive only in microtube 1 where a change of colour from yellow to red was observed, and the suspensions in microtubes 2 and 3 remained yellow, (iii) isolates producing a carbapenemase, including MBLs, inhibited by TAN were positive in microtubes 1 and 2 (red colour), but not in 3 (yellow), and (iv) isolates carrying a carbapenemase, including MBLs, not inhibited by TAN were positive in all the suspensions (all microtubes changed from yellow to red) (Fig. 1). All the results were obtained and interpreted within 15 min of incubation at room temperature, and results were blindly read and interpreted independently by two laboratory members. Of note, the test proposed here could be used for screening any new compounds supposed to be potential β-lactamase inhibitors by simply replacing the TAN by such molecule.

## Results and discussion

The NitroSpeed Taniborbactam NP test demonstrated excellent agreement with the genotypic characterization of the strains, accurately distinguishing β-lactamase production inhibited by TAN (e.g. NDM-1, VIM-2, GES-5) from those not inhibited (e.g. IMPs, NDM-9, VIM-83). A total of 95 (70.9%) strains produced β-lactamases that were inhibited by TAN, while 39 (29.1%) strains harboured enzymes that were not susceptible to TAN inhibition. Using the presence of these enzymes as the reference gold standard, the test achieved 100% sensitivity, specificity, and accuracy with no false-positive or false-negative results obtained. The complete results are shown in Table 1 and Supplementary Table 1.

Despite its limited activity against certain MBLs, TAN exhibits remarkable β-lactamase inhibitory activity against some of the most globally prevalent carbapenemases such as KPC, and MBLs such as NDM-1 and VIM-2 (Bosch et al. 2017, Pirzadian et al. 2021). When combined with cefepime, TAN is poised to become the first clinically available MBL inhibitor, initially indicated for the treatment of complicated urinary tract infections (UTIs). The optimal and appropriate use of TAN is crucial for efficiently treating patients while mitigating the risk of selecting for resistant variants.

In phase III clinical trials (NCT03840148), the cefepime–TAN combination has demonstrated superior efficacy compared to meropenem in patients with complicated UTIs, showing a safety profile comparable to carbapenems (Wagenlehner et al. 2024). Given these promising results, we hypothesize that TAN-related molecules or other boronate inhibitors may exhibit similar or even superior activity against resistant MBLs. According to Drusin et al. (2024), IMP-type MBLs and other enzymes less susceptible to TAN inhibition possess more neutral-to-positively charged active sites, whereas NDM-1 and VIM-2 exhibit highly negatively charged active sites. Thus, designing novel β-lactamase inhibitors should focus on electrostatic interactions within the active site loops L2 and L10 of the MBLs., optimizing binding affinity and inhibitory efficacy.

We have developed the NitroSpeed Taniborbactam NP test as a rapid and reliable tool for assessing the inhibitory activity of TAN against MBLs and other carbapenemases. Furthermore, this test provides a screening method for identifying new inhibitors that could be effective against MBLs naturally resistant to TAN such as IMPs, NDM-9, NDM-30, VIM-83, and SIM-1. The test is simple, easy to perform, and yields results within 15 min. Importantly, in cases where reduced test accuracy may arise potentially due to altered enzymatic kinetics of β-lactamases, the test conditions could be adjusted accordingly, reflecting its adaptable nature.

A key limitation of the NitroSpeed Taniborbactam NP test is that while it specifically detects enzymatic resistance to TAN, it does not account for non-enzymatic resistance mechanisms such as outer membrane permeability defects or overexpression of efflux pumps. Having successfully passed its initial phase of evaluation, the test will now enter a second phase involving a broader and more diverse set of microorganisms. This phase aims to determine whether the test can be extrapolated to detect susceptibility or resistance in combination with cefepime.

The development of future inhibitors will depend either on chemical modifications of existing inhibitors or on the discovery of entirely new inhibitors. In this context, the NitroSpeed Taniborbactam NP test could serve as a valuable, cell-based assay for evaluating β-lactamase-expressing clinical strains, contributing to the advancement of novel β-lactamase inhibitors.

## Supplementary Material

fnaf044_Supplemental_File
