# Supplementary material for: The NitroSpeed Taniborbactam NP test as a rapid test for detection of β-lactamase-mediated susceptibility to taniborbactam
Source: FEMS Microbiol Lett. 2025 May 6;372:fnaf044. doi: 10.1093/femsle/fnaf044 (PMC12065395; doi:10.1093/femsle/fnaf044)
Supplement: fnaf044_Supplemental_File [file fnaf044_supplemental_file.docx]

Supplementary Table 1. NitroSpeed Taniborbactam test for the detection of β-lactamases enzymatic activity inhibited or not by Taniborbactam in Enterobacterales and *Pseudomonas aeruginosa*

| Strain number | Species | Ambler Class | Main acquired β-lactamase | NitroSpeed Taniborbactam test | | |
| --- | --- | --- | --- | --- | --- | --- |
|  |  |  |  | Water | ETP | ETP + TAN |
| 1 | *E. coli* ATCC 25922 | NA | NA | Y | Y | Y |
| 2 | *E. coli* | NA | AmpC overproduction | R | Y | Y |
| 3 | *E. coli* | A | CTX-M-1 | R | Y | Y |
| 4 | *K. pneumoniae* | A | CTX-M-15 | R | Y | Y |
| 5 | *C. freundii* | A | CTX-M-15 | R | Y | Y |
| 6 | *E. cloacae* | A | TEM-1 | R | Y | Y |
| 7 | *K. pneumoniae* | A | TEM-1 | R | Y | Y |
| 8 | *E. cloacae* | A | VEB-1 | R | Y | Y |
| 9 | *E. cloacae* | A | VEB-1 | R | Y | Y |
| 10 | *K. pneumoniae* | A | KPC-2 | R | R | Y |
| 11 | *E. coli* | B | NDM-1 | R | R | Y |
| 12 | *E. coli* | B | NDM-1 | R | R | Y |
| 13 | *E. coli* | B | NDM-1 | R | R | Y |
| 14 | *E. coli* | B | NDM-1 | R | R | Y |
| 15 | *K. pneumoniae* | B | NDM-1 | R | R | Y |
| 16 | *K. pneumoniae* | B | NDM-1 | R | R | Y |
| 17 | *K. pneumoniae* | B | NDM-1 | R | R | Y |
| 18 | *K. pneumoniae* | B | NDM-1 | R | R | Y |
| 19 | *K. pneumoniae* | B | NDM-1 | R | R | Y |
| 20 | *K. pneumoniae* | B | NDM-1 | R | R | Y |
| 21 | *K. pneumoniae* | B | NDM-1 | R | R | Y |
| 22 | *K. pneumoniae* | B | NDM-1 | R | R | Y |
| 23 | *K. pneumoniae* | B+D | NDM-1 + OXA-48 | R | R | Y |
| 24 | *K. pneumoniae* | B+D | NDM-1 + OXA-48 | R | R | Y |
| 25 | *K. pneumoniae* | B+D | NDM-1 + OXA-181 | R | R | Y |
| 26 | *K. oxytoca* | B | NDM-1 | R | R | Y |
| 27 | *C. freundii* | B | NDM-1 | R | R | Y |
| 28 | *C. freundii* | B | NDM-1 | R | R | Y |
| 29 | *C. freundii* | B | NDM-1 | R | R | Y |
| 30 | *C. freundii* | B | NDM-1 | R | R | Y |
| 31 | *E. cloacae* | B | NDM-1 | R | R | Y |
| 32 | *E. cloacae* | B | NDM-1 | R | R | Y |
| 33 | *E. cloacae* | B | NDM-1 | R | R | Y |
| 34 | *E. coli* | B | NDM-4 | R | R | Y |
| 35 | *E. coli* | B | NDM-4 | R | R | Y |
| 36 | *E. coli* | B | NDM-5 | R | R | Y |
| 37 | *E. coli* | B | NDM-5 | R | R | Y |
| 38 | *E. coli* | B | NDM-5 | R | R | Y |
| 39 | *E. coli* | B | NDM-5 | R | R | Y |
| 40 | *E. coli* | B | NDM-5 | R | R | Y |
| 41 | *E. coli* | B | NDM-5 | R | R | Y |
| 42 | *E. coli* | B | NDM-5 | R | R | Y |
| 43 | *E. coli* | B+D | NDM-5 + OXA-181 | R | R | Y |
| 44 | *E. coli* | B | NDM-7 | R | R | Y |
| 45 | *K. pneumoniae* | B | NDM-7 | R | R | Y |
| 46 | *C. freundii* | B | NDM-7 | R | R | Y |
| 47 | *E. cloacae* | B | NDM-7 | R | R | Y |
| 48 | *E. coli* | B | **NDM-9** | **R** | **R** | **R** |
| 49 | *E. coli* | B | **NDM-9** | **R** | **R** | **R** |
| 50 | *K. pneumoniae* | B | **NDM-9** | **R** | **R** | **R** |
| 51 | *K. pneumoniae* | B | **NDM-9** | **R** | **R** | **R** |
| 52 | *K. variicola* | B | **NDM-9** | **R** | **R** | **R** |
| 53 | *K. variicola* | B | **NDM-9** | **R** | **R** | **R** |
| 54 | *K. variicola* | B | **NDM-9** | **R** | **R** | **R** |
| 55 | *E. coli* | B | NDM-19 | R | R | Y |
| 56 | *E. coli* | B | **NDM-30** | **R** | **R** | **R** |
| 57 | *E. coli* | B | VIM-1 | R | R | Y |
| 58 | *E. coli* | B | VIM-1 | R | R | Y |
| 59 | *E. coli* | B | VIM-1 | R | R | Y |
| 60 | *K. pneumoniae* | B | VIM-1 | R | R | Y |
| 61 | *K. pneumoniae* | B | VIM-1 | R | R | Y |
| 62 | *K. pneumoniae* | B | VIM-1 | R | R | Y |
| 63 | *K. pneumoniae* | B | VIM-1 | R | R | Y |
| 64 | *K. pneumoniae* | B | VIM-1 | R | R | Y |
| 65 | *K. pneumoniae* | B | VIM-1 | R | R | Y |
| 66 | *K. pneumoniae* | B | VIM-1 | R | R | Y |
| 67 | *K. pneumoniae* | B | VIM-1 | R | R | Y |
| 68 | *E. cloacae* | B | VIM-1 | R | R | Y |
| 69 | *E. cloacae* | B | VIM-1 | R | R | Y |
| 70 | *E. cloacae* | B | VIM-1 | R | R | Y |
| 71 | *E. coli* | B | VIM-2 | R | R | Y |
| 72 | *C. freundii* | B | VIM-2 | R | R | Y |
| 73 | *C. freundii* | B | VIM-2 | R | R | Y |
| 74 | *C. freundii* | B | VIM-2 | R | R | Y |
| 75 | *C. freundii* | B | VIM-2 | R | R | Y |
| 76 | *C. freundii* | B | VIM-2 | R | R | Y |
| 77 | *C. freundii* | B | VIM-2 | R | R | Y |
| 78 | *C. freundii* | B | VIM-2 | R | R | Y |
| 79 | *E. cloacae* | B | VIM-4 | R | R | Y |
| 80 | *E. coli* | B | **VIM-83** | **R** | **R** | **R** |
| 81 | *E. coli* | B | **IMP-1** | **R** | **R** | **R** |
| 82 | *K. pneumoniae* | B | **IMP-1** | **R** | **R** | **R** |
| 83 | *C. freundii* | B | **IMP-1** | **R** | **R** | **R** |
| 84 | *K. pneumoniae* | B | **IMP-4** | **R** | **R** | **R** |
| 85 | *C. freundii* | B | **IMP-4** | **R** | **R** | **R** |
| 86 | *E. hormaechei* | B | **IMP-4** | **R** | **R** | **R** |
| 87 | *E. hormaechei* | B | **IMP-4** | **R** | **R** | **R** |
| 88 | *E. coli* | B | **IMP-6** | **R** | **R** | **R** |
| 89 | *K. pneumoniae* | B | **IMP-6** | **R** | **R** | **R** |
| 90 | *K. pneumoniae* | B | **IMP-6** | **R** | **R** | **R** |
| 91 | *E. hormaechei* | B | **IMP-8** | **R** | **R** | **R** |
| 92 | *E. hormaechei* | B | **IMP-8** | **R** | **R** | **R** |
| 93 | *S. marcescens* | B | **IMP-8** | **R** | **R** | **R** |
| 94 | *Escherichia coli* | B | **IMP-14** | **R** | **R** | **R** |
| 95 | *K. pneumoniae* | B | **IMP-14** | **R** | **R** | **R** |
| 96 | *E. hormaechei* | B | **IMP-14** | **R** | **R** | **R** |
| 97 | *K. pneumoniae* | B | **IMP-26** | **R** | **R** | **R** |
| 98 | *K. pneumoniae* | B | **IMP-26** | **R** | **R** | **R** |
| 99 | *K. pneumoniae* | B | **IMP-34** | **R** | **R** | **R** |
| 100 | *E. coli* | B | **IMP-59** | **R** | **R** | **R** |
| 101 | *E. coli* | B | **IMP-59** | **R** | **R** | **R** |
| 102 | *E. coli* | B | **SIM-1** | **R** | **R** | **R** |
| 103 | *E. cloacae* | D | OXA-48 | R | R | Y |
| 104 | *P. aeruginosa* ATCC 27853 | NA | NA | Y | Y | Y |
| 105 | *P. aeruginosa* PAO1 | NA | NA | Y | Y | Y |
| 106 | *P. aeruginosa* | NA | PDC overproduction | R | Y | Y |
| 107 | *P. aeruginosa* | NA | PDC overproduction | R | Y | Y |
| 108 | *P. aeruginosa* | A | GES-5 | R | R | Y |
| 109 | *P. aeruginosa* | A | VEB-1 | R | Y | Y |
| 110 | *P. aeruginosa* | A | VEB-9 | R | Y | Y |
| 111 | *P. aeruginosa* | A | KPC-2 | R | R | Y |
| 112 | *P. aeruginosa* | B | NDM-1 | R | R | Y |
| 113 | *P. aeruginosa* | B | NDM-1 | R | R | Y |
| 114 | *P. aeruginosa* | B | NDM-1 | R | R | Y |
| 115 | *P. aeruginosa* | B | NDM-1 | R | R | Y |
| 116 | *P. aeruginosa* | B | NDM-1 | R | R | Y |
| 117 | *P. aeruginosa* | B | NDM-1 | R | R | Y |
| 118 | *P. aeruginosa* | B | NDM-1 | R | R | Y |
| 119 | *P. aeruginosa* | B | VIM-1 | R | R | Y |
| 120 | *P. aeruginosa* | B | VIM-2 | R | R | Y |
| 121 | *P. aeruginosa* | B | VIM-4 | R | R | Y |
| 122 | *P. aeruginosa* | B | VIM-4 | R | R | Y |
| 123 | *P. aeruginosa* | B | VIM-5 | R | R | Y |
| 124 | *P. aeruginosa* | B | VIM-5 | R | R | Y |
| 125 | *P. aeruginosa* | B | VIM-36 | R | R | Y |
| 126 | *P. aeruginosa* | B | **IMP-1** | **R** | **R** | **R** |
| 127 | *P. aeruginosa* | B | **IMP-1** | **R** | **R** | **R** |
| 128 | *P. aeruginosa* | B | **IMP-1** | **R** | **R** | **R** |
| 129 | *P. aeruginosa* | B | **IMP-1** | **R** | **R** | **R** |
| 130 | *P. aeruginosa* | B | **IMP-1** | **R** | **R** | **R** |
| 131 | *P. aeruginosa* | B | **IMP-7** | **R** | **R** | **R** |
| 132 | *P. aeruginosa* | B | **IMP-7** | **R** | **R** | **R** |
| 133 | *P. aeruginosa* | B | **IMP-13** | **R** | **R** | **R** |
| 134 | *P. aeruginosa* | B | SPM-1 | R | R | Y |

NA, not applicable; (Y), Yellow color: enzyme is inhibited by Taniborbactam ; (R), Red color: enzyme is not inhibited by Taniborbactam; Bold script, enzymes not inhibited by Taniborbactam
